# Comparing the beliefs regarding biological or psychological causalities toward stereotyped perception of people who stutter

**DOI:** 10.3389/fpsyg.2023.1279169

**Published:** 2023-11-16

**Authors:** Daichi Iimura, Osamu Ishida

**Affiliations:** ^1^Institute of Human Sciences, University of Tsukuba, Ibaraki, Japan; ^2^College of Education, Ibaraki University, Ibaraki, Japan

**Keywords:** stuttering, causality, stereotyping, surveys and questionnaires, perception

## Abstract

**Purpose:**

Developmental stuttering is a fluency disorder that may be caused by neurological, genetic, or familial factors. However, a general perception that stuttering is caused by psychological problems could lead to negative attitudes toward stuttering, causing prejudice or discrimination against people who stutter (PWS). Thus, our study aimed to investigate whether certain beliefs in etiology of stuttering are related to the negative perception of stuttering.

**Methods:**

A web-based survey of 413 native Japanese adults, aged 20−69, who did not suffer from stuttering, schizophrenia, or depression, was conducted in August 2021. The participants were recruited through the Web monitor panel. Participants were divided into three uniform groups based on their response to a 27-item questionnaire about their implicit belief regarding the etiology of stuttering: belief in the biological model (stuttering-biological group), belief in the psychological model (stuttering-psychological group), and the control group (those who responded to perception of healthy adult males). Participants were also asked to respond to 25 items of semantic differential scales about perception of stuttering or healthy adult males. Responses were summarized into several factors by factor analysis, and factor scores were compared among the three groups. The stuttering-biological group had the fewest participants, comprising 80 individuals. Overall, a total of 240 participants, 80 from each group, were included in the analysis.

**Results:**

Some pairs of stereotypes included in semantic differential scales revealed differences between the groups; PWS, irrespective of the participants of the biological or psychological group, were considered as having negative stereotyping properties such as being “tense,” “anxious,” or “afraid.” Additionally, three concepts from the factor analysis of these 25 items were analyzed using an analysis of variance, and significant differences were found; the mean factor score of the “danger” stereotype was lower in the stuttering-biological group compared to the stuttering-psychological group.

**Conclusion:**

Although the simplification of the biological model is not recommended, anti-stigma campaigns to educate people that stuttering is caused by multidimensional factors, not just psychological ones, could change the general public’s negative perceptions of stuttering.

## 1. Introduction

Developmental stuttering is a fluency disorder characterized by frequent word or part-word repetition, prolongation, or silent blocks that disrupt the rhythmic flow of speech (e.g., Guitar, 2018). Genetic factors have been proposed as a possible cause (Kraft and Yairi, 2012; Chang and Zhu, 2013; Frigerio-Domingues and Drayna, 2017). Several studies have found negative stereotyped perceptions of people who stutter (PWS) among the general population (e.g., St Louis and Tellis, 2015; Boyle, 2016a; Amick et al., 2017; Byrd et al., 2017). Stereotypes regarding stuttering were first discussed about half a century ago (Yairi and Williams, 1970; Woods and Williams, 1971, 1976; St Louis et al., 2017). A semantic differential (SD) scale comprising 25 paired items by Woods and Williams (1976) has been used often in the recent literature on stuttering stereotypes (Collins and Blood, 1990; Klassen, 2001, 2002; Gabel, 2006; Betz et al., 2008; Boyle et al., 2009; Hughes et al., 2017). These studies refer to the belief that PWS are perceived by the general public as being nervous, shy, reticent, passive, fearful, etc., as opposed to self-confident, outgoing, friendly, and otherwise attractive to others.

Numerous studies have focused on the negative perceptions of individuals with mental disorders. In particular, the belief regarding the etiology of specific disorders tends to affect one’s perceptions (i.e., Weiner, 1995; Menec and Perry, 1998; Phelan, 2002), especially of depression (Goldstein and Rosselli, 2003; Breheny, 2007; Rusch et al., 2009; Nieuwsma and Pepper, 2010) and schizophrenia (Breheny, 2007; Bennett et al., 2008; Lincoln et al., 2008). Goldstein and Rosselli’s (2003) factor analysis produced three distinct models of the etiology: biological, psychological, and environmental. Regression analyses showed that endorsement of the biological model was associated with increased empowerment, preference for psychotherapy, and decreased stigma, whereas endorsement of the psychological model was associated with increased stigma and an increased belief that people can help themselves. These findings were supported by genetic attribution theory (Weiner et al., 1988; Weiner, 1995; Corrigan and Watson, 2002; Corrigan and Shapiro, 2010). Weiner (1995) illustrated that attributing personal responsibility for a controllable adverse event leads to anger and diminishes helping behavior. Conversely, attributing no blame for a harmful event which is not controllable leads to pity and the desire to help (Corrigan and Watson, 2002; Corrigan and Shapiro, 2010). As with depression, controllable disorders could provoke greater stigmatization, while the biological model might serve to reduce stigma by eliminating the belief that depression is controllable (Goldstein and Rosselli, 2003). Stigma plays a roles in viewing this as a major public health issue (Shalbafan et al., 2023), and interventions that decrease the public-stigma (e.g., Taghva et al., 2022).

As stuttering is a concern for a minority of an overall population (approximately 1%) (Yairi and Ambrose, 2013) and PWS try to conceal their stuttering when interacting with other people (Constantino et al., 2017; Boyle and Gabel, 2020), the general public rarely observes stuttering behaviors. This could widen the social distance between the general public and PWS. Owing to this, the general public could over-generalize any one aspect of a stuttering person—which leads to stereotyping of PWS. The controllability of stuttering is associated with people’s attitudes toward stuttering. Focusing on the negative stereotypes of stuttering (e.g., stuttering is a psychological matter that can be controlled for oneself) could lead to greater stigmatization.

To assess the stereotyped perception of stuttering, the SD scale of Woods and Williams (1976) has been replicated to investigate variables such as attending stuttering therapy, stuttering severity (Gabel, 2006), explicit causality of stuttering (Boyle et al., 2009), familiarity with PWS (Klassen, 2001; Betz et al., 2008; Hughes et al., 2017), and PWS’ acknowledgment of their stuttering (Collins and Blood, 1990). For example, Gabel (2006) found that more positive characteristics were associated with males who had mild stuttering compared to those who had severe stuttering, and with males who attended therapy to improve their fluency compared to those who did not. In Boyle et al.’s (2009) study, university students rated psychological causes more negatively on 14 adjective pairs than they rated genetic or unknown causes through vignette descriptions. However, it remains unclear whether the stereotypes of stuttering could differ by an individual’s belief regarding the etiology of stuttering as biological or psychological. The impact of the etiology of certain disorders has already been investigated in studies on mental disorders (Weiner, 1995; Menec and Perry, 1998; Phelan, 2002; Goldstein and Rosselli, 2003; Breheny, 2007; Bennett et al., 2008; Lincoln et al., 2008; Rusch et al., 2009; Nieuwsma and Pepper, 2010), but few studies on stuttering have addressed this issue (Boyle et al., 2009, 2016). Although the associations may be similar in the case of stuttering, no studies have yet measured one’s implicit belief regarding the etiology of stuttering.

The present study investigated whether certain beliefs regarding the etiology of stuttering are related to the negative perceptions of stuttering. Our study is novel in elucidating the association between implicit belief regarding the etiology of stuttering and the negative perceptions of stuttering. Advancements have been made in the etiology of stuttering, and the findings could contribute to an anti-stigma campaign on stuttering, the importance of which has been well emphasized (e.g., Boyle et al., 2016, 2017; Boyle, 2017).

## 2. Materials and methods

### 2.1. Participants

Participants were 413 adults who were registered with the Macromill monitor panel (Macromill, Inc., Tokyo, Japan). In total, 1.2 million people, aged 20−69, who were registered with the panel accounted for approximately 1.6% of this population in Japan. Inclusion criteria were: (1) 20−69 years of age; (2) understand Japanese; and (3) do not have stuttering, schizophrenia, or depression. Of these monitor panels, a stratified sample of 2 × 5 cells was determined by gender (male or female) and age (20, 30, 40, 50, or 60s). Monitor participants belonging to each cell were randomly sent a message from Macromill Inc., via e-mail or web app in August 2021, and participants accessed the survey through the link provided. The survey was open until the scheduled number of participants was reached. Participants who responded to the survey received rewards that were exchangeable for cash after survey completion.

We obtained informed consent from each participant after confirming their voluntary participation. The research ethics committee of the Kawasaki University of Medical Welfare (20-092) (previous affiliation of the first author) approved the study’s experimental procedures in advance.

### 2.2. Design

**FIGURE 1:**
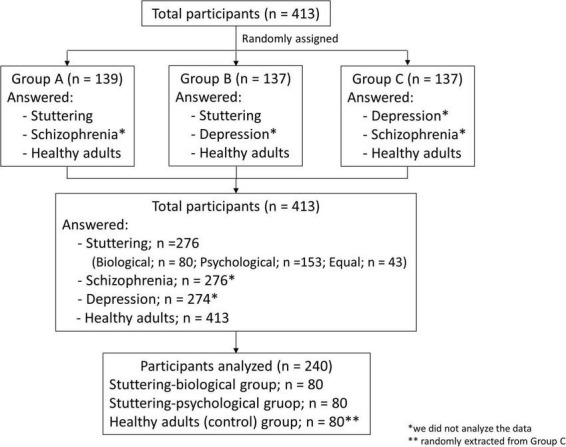
Flowchart demonstrating the group assignment of participants.

The investigation is part of the extensive survey comprising ten question lists that investigate the general population’s perceptions and attitudes toward stuttering and two other mental disorders. Participants were assigned randomly to answer questions about their perceptions of two of the three specific disorders: males with stuttering, schizophrenia, and depression (Figure 1). All participants were also asked to respond regarding their perceptions of healthy adults. In total, 139 participants answered questions about stuttering, schizophrenia, and healthy adults (Group A), 137 participants answered about stuttering, depression, and healthy adults (Group B), and 137 participants answered about schizophrenia, depression, and healthy adults (Group C). As a result, 276 participants answered questions about stuttering, and 413 answered about healthy adults. The present study did not analyze data on schizophrenia and depression question lists.

The study intended to compare perceptions of stuttering based on participants’ beliefs regarding the etiology of stuttering. Participants were grouped using mean factor scores of items that examined their implicit beliefs regarding the etiology of stuttering (questionnaire description in section “2.3.1. Belief regarding the etiology of stuttering”). We compared each group’s responses to the questionnaire of stereotyped perception (questionnaire description in section “2.3.2. Stereotyped perception of adult males with stuttering and healthy adult males) in the section “3. Results.”

### 2.3. Measures

The multiple-choice questionnaire used in the study comprised question lists on (1) belief regarding the etiology of stuttering, (2) stereotyped perception of males with stuttering, and (3) stereotyped perception of healthy adult males. We provided brief information regarding stuttering beforehand: “stuttering disrupts fluent speech due to the following symptoms: repetition of the initial words (a, a, apple), prolongation (e.g., a–pple), and blocks (e.g., apple).” Redundant descriptions could have a bias that influences one’s beliefs regarding stuttering; hence, brief descriptions of the three core stuttering symptoms (Guitar, 2018) were provided.

#### 2.3.1. Belief regarding the etiology of stuttering

To measure participants’ implicit beliefs regarding the etiology of stuttering, a total of 27 items were prepared through discussions of two authors by selecting and combining items based on previous findings; literature on stuttering (de Britto Pereira et al., 2008; St Louis, 2011; Iimura et al., 2018), depression, or schizophrenia (Goldstein and Rosselli, 2003; Lincoln et al., 2008; Nieuwsma and Pepper, 2010). Participants were required to evaluate their belief about the etiology of stuttering on 27 items (i.e., brain disease, general stress, etc.) using a 7-point Likert-scale ranging from “certainly not a cause (scored 1)” to “certainly a cause (scored 7).” The order of the 27 items was randomized across participants.

#### 2.3.2. Stereotyped perception of adult males with stuttering and healthy adult males

Stereotyped perceptions were assessed using the SD scale of Woods and Williams (1976), which comprises 25 bi-polar adjectives regarding various characteristics (e.g., open-guarded, tense-relaxed, etc.). Woods and Williams (1976) showed that most items (23 out of the 25) were significantly different between stuttering males and those speaking normally, with the former regarded as “tense,” “withdrawn,” “quiet,” and so on. On a 7-point Likert scale, participants were required to evaluate the characteristics of males with stuttering, schizophrenia, or depression (e.g., participants in Group A answered about stuttering males and males with schizophrenia; see Figure 1). All participants were also required to evaluate healthy adult males. The order of the 25 items was randomized among the participants.

Since the original questionnaires (described in section “2.3.1. Belief regarding the etiology of stuttering” and section “2.3.2. Stereotyped perception of adult males with stuttering and healthy adult males”) were developed in English, the scales were translated through the following process to ensure translation reliability. First, English phrases were translated into Japanese by the first author, a native Japanese speaker. The draft was back-translated into English by a trained translator.^1^ Thereafter, the first and second authors carefully compared the original and back-translated questionnaires written in English. Finally, the two authors confirmed that all English words within the back-translated version were retained.

### 2.4. Procedures

Participants were required to respond to all the questions via the Macromill Inc., website. To determine the groups based on their beliefs regarding the etiology of stuttering, we performed a factor analysis of 27 items that examined their implicit beliefs regarding the etiology of stuttering (section “2.3.1. Belief regarding the etiology of stuttering”). Mean, standard deviation, and range of participants are shown in Table 1, with ceiling or floor effects not observed. The KMO (Kaiser-Meyer-Olkin measure of sampling adequacy) value was found to be 0.939, showing that the data were fit for factor analysis. Table 2 shows the results of factor analysis of the least-squares regression models with promax rotation. In the first round of factor analysis, we used the widely accepted Kaiser (1960) method that adapted factors with eigenvalues greater than 1. Therefore, a three-factor structure was adopted first: biological model (Factor 1; 7 items), psychological model (Factor 2; 10 items), and others (Factor 3; 10 items). Considering the deliberations regarding the biological and psychological models on stuttering in preceding studies (Boyle et al., 2009, 2016), the 10 items of Factor 3 were eliminated. Two items included in Factor 2 (i.e., “problematic childhood” and “environmental factors”) were also eliminated because these were regarded as environmental factors.

**TABLE 1:** Mean, standard deviation, and range of participants of the 27 items of the etiology of stuttering.

	Mean	Standard deviation	Range
brain disease (transmitter disorder and morphological anomalies)	4.601	1.671	1.00−7.00
inheritance/genetics	4.391	1.645	1.00−7.00
biological changes in brain	4.257	1.493	1.00−7.00
brain damage (e.g., poisoning or injuries)	4.533	1.648	1.00−7.00
biochemical abnormalities	3.696	1.509	1.00−7.00
chemical/hormone imbalance	3.888	1.462	1.00−7.00
biology	3.924	1.534	1.00−7.00
general stress	4.837	1.675	1.00−7.00
stressors and strain	4.960	1.729	1.00−7.00
traumatic event	4.594	1.710	1.00−7.00
psychogenic	4.793	1.597	1.00−7.00
problematic childhood (e.g., unloving parents, too strict, or inconsequent upbringing)	4.362	1.707	1.00−7.00
negative life event	4.101	1.608	1.00−7.00
environmental factors	4.337	1.579	1.00−7.00
recent misfortunes	3.902	1.594	1.00−7.00
emotional	4.167	1.641	1.00−7.00
self-induced (e.g., weak will, impulsiveness, or immoral behavior)	3.848	1.627	1.00−7.00
ghosts, demons, spirits	2.348	1.594	1.00−7.00
God’s will (e.g., punishment or test)	2.417	1.583	1.00−7.00
lack of will power	3.543	1.505	1.00−7.00
poor cognitive outlook	3.301	1.504	1.00−7.00
learned helplessness	3.489	1.548	1.00−7.00
personality	3.598	1.632	1.00−7.00
melancholic personality	3.859	1.553	1.00−7.00
faulty learning or habits	3.754	1.662	1.00−7.00
low (lack of) social support	3.826	1.638	1.00−7.00
expecting too much of self	3.761	1.526	1.00−7.00

**TABLE 2:** First round of the factor analysis on the 27 items of the etiology of stuttering.

	Factor 1 Biological model	Factor 2 Psychological model	Factor 3 Others	Communality
brain disease (transmitter disorder and morphological anomalies)	**0.790**	0.171	−0.197	0.674
inheritance/genetics	**0.762**	−0.018	0.019	0.577
biological changes in brain	**0.712**	0.118	−0.043	0.602
brain damage (e.g., poisoning or injuries)	**0.523**	0.302	0.001	0.587
biochemical abnormalities	**0.495**	−0.089	0.390	0.516
chemical/hormone imbalance	**0.476**	0.066	0.350	0.606
biology	**0.398**	0.139	0.308	0.532
general stress	−0.011	**0.916**	−0.121	0.716
stressors and strain	0.072	**0.913**	−0.280	0.700
traumatic event	−0.024	**0.905**	−0.062	0.732
psychogenic	0.106	**0.839**	−0.166	0.692
*problematic childhood (e.g., unloving parents, too strict, or inconsequent upbringing)	0.117	**0.787**	−0.143	0.637
negative life event	−0.012	**0.628**	0.234	0.601
*environmental factors	0.055	**0.564**	0.150	0.490
recent misfortunes	0.057	**0.528**	0.246	0.545
emotional	0.086	**0.525**	0.160	0.482
self-induced (e.g., weak will, impulsiveness, or immoral behavior)	0.051	**0.430**	0.379	0.567
*ghosts, demons, spirits	0.036	−0.489	**0.857**	0.513
*God’s will (e.g., punishment or test)	0.031	−0.458	**0.830**	0.481
*lack of will power	−0.077	0.201	**0.725**	0.653
*poor cognitive outlook	0.011	0.038	**0.702**	0.532
*learned helplessness	−0.065	0.198	**0.700**	0.621
*personality	−0.078	0.262	**0.631**	0.577
*melancholic personality	0.010	0.278	**0.615**	0.659
*faulty Learning or habits	−0.006	0.332	**0.475**	0.506
*low (lack of) social support	−0.031	0.367	**0.468**	0.517
*expecting too much of self	−0.060	0.408	**0.451**	0.516

* Item excluded from further analysis. Least-squares regression models with promax rotation.

In the second round of factor analysis of 15 items, the two-factor structure was found: biological (Factor 1; 8 items) and psychological models (Factor 2; 7 items). One item (“self-induced”) included in Factor 1 was excluded, because it was considered in Factor 2. Thereafter, the third round of factor analysis that included 14 items yielded results similar to the second round: biological model (7 items) and psychological model (7 items) (Table 3). The factor analysis was completed in this round as it was confirmed that the two factors, both with eigenvalues loadings greater than 1, consisted of the “biological model” and “psychological model.” The reliability of internal consistency was verified as acceptable (Table 4).

**TABLE 3:** Final round of the factor analysis on the 14 items of the etiology of stuttering.

	Factor 1 Biological model	Factor 2 Psychological model	Communality
biochemical abnormalities	**0.798**	−0.058	0.574
inheritance/genetics	**0.792**	−0.133	0.494
chemical/hormone imbalance	**0.739**	0.033	0.583
biology	**0.700**	0.037	0.528
biological changes in brain	**0.661**	0.116	0.561
brain disease (transmitter disorder and morphological anomalies)	**0.605**	0.168	0.540
brain damage (e.g., poisoning or injuries)	**0.495**	0.318	0.572
stressors and strain	−0.108	**0.923**	0.720
general stress	−0.064	**0.903**	0.736
traumatic event	−0.012	**0.868**	0.738
psychogenic	0.014	**0.831**	0.707
negative life event	0.272	**0.505**	0.527
recent misfortunes	0.281	**0.485**	0.510
emotional	0.305	**0.426**	0.460

Least-squares regression models with promax rotation.

**TABLE 4:** Reliability statistics for questionnaires.

	Factor	Internal consistency (Cronbach’s α)
Etiology of three factors (Table 2)	Biological	0.933
	Psychological	0.904
	Other	0.892
Etiology of two factors (Table 3)	Biological	0.916
	Psychological	0.892
Stereotypes questionnaire (Table 6)	Factor 1	0.908
	Factor 2	0.771
	Factor 3	0.735
	Factor 4	0.339
	Factor 5	0.142

### 2.5. Data cleaning

For the 276 participants comprising Groups A and B (Figure 1), we compared the mean score of items which were included in Factors 1 and 2. The factor with the higher mean score was considered the etiology of stuttering as believed by the participants; 80 participants were regarded as the stuttering-biological group. In total, 153 participants were regarded as the stuttering-psychological group, and 80 participants were selected randomly from this group to create the same sample size. Forty-three participants had identical mean scores between Factors 1 and 2 and were thus excluded from further analysis. To form the control group (i.e., participants who responded regarding healthy adults), 80 participants were selected randomly from Group C. As all questions had to be answered to complete the survey, no data were missing for any of the participants.

### 2.6. Data analysis

Responses to the questionnaire on stereotyped perception (section “2.3.2. Stereotyped perception of adult males with stuttering and healthy adult males”) were statistically compared among the three groups: stuttering-biological group, stuttering-psychological group, and healthy adults (control) group. The responses to the 25 bipolar items of stereotyped perception (section “2.3.2. Stereotyped perception of adult males with stuttering and healthy adult males”) were analyzed by comparing the mean score of three groups. Items were summarized into a few concepts through factor analysis. Each participant’s factor scores were then calculated and analyzed by a two-factor mixed-design ANOVA with Shaffer’s multiple comparisons. One factor is the between-participants factor of three levels (Group: Stuttering-biological vs. Stuttering-psychological vs. Control), and another is the within-participants factor (characteristics found by factor analysis).

## 3. Results

### 3.1. Sample demographics after cleaning

Participants’ demographic information is shown in Table 5. Each group consisted of 80 participants. There was no significant difference in the distribution of the three groups regarding demographic information (chi-square analysis; *p* > 0.05), except for the age distribution of participants in their 20s, which had a small effect [χ2 (8) = 16.10, *p* = 0.041, Cramer’s V = 0.18]. Thus, both groups were considered demographically homogeneous.

**TABLE 5:** Demographics of participants (*n* = 240).

		Total *N* (%)	N of stuttering (biological) group	N of stuttering (psychological) group	N of control group
**Age (years)**					
	20s 30s 40s 50s 60s	28 (11.7%) 53 (22.1%) 49 (20.4%) 55 (22.9%) 55 (22.9%)	11 17 19 16 17	14* 23 14 15 14	3** 13 16 24 24
**Gender**					
	Male Female	135 (56.3%) 105 (43.7%)	50 30	38 42	47 33
**Highest educational level**					
	Junior high school High school Specialized vocational high school Junior college or vocational college University Graduate school	4 (1.7%) 59 (24.6%) 8 (3.3%) 47 (19.6%) 111 (46.3%) 11 (4.6%)	2 19 3 16 35 5	1 20 3 15 38 3	1 20 2 16 38 3
**Married**					
	Yes No	165 (68.7%) 75 (31.3%)	55 25	51 29	59 21
**Have a child**					
	Yes No	105 (43.8%) 135 (56.2%)	34 46	40 40	31 49
**Employment**					
	Yes No Unknown	148 (61.7%) 59 (24.6%) 5 (2.1%)	62 17 1	58 19 3	56 23 1

**p < 0.01. *p < 0.05.

### 3.2. Mean scores of the questionnaire

**FIGURE 2:**
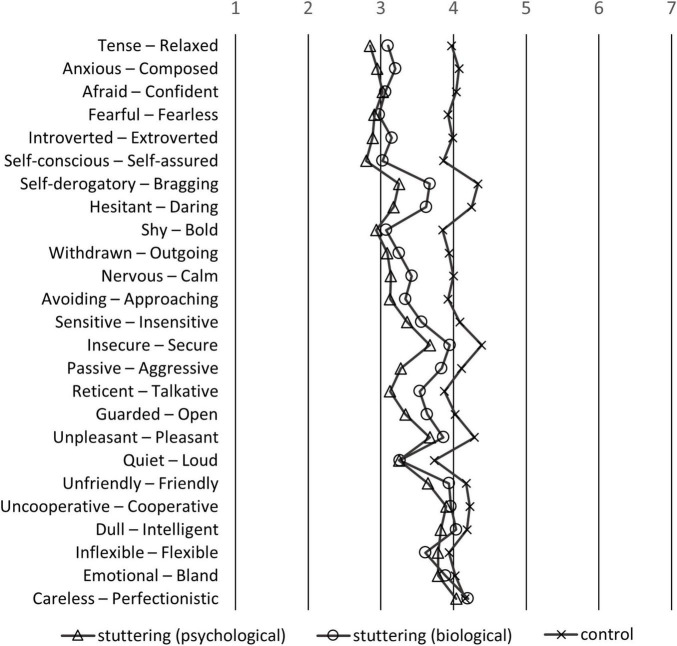
Means of the participants of the stereotypes questionnaire sorted by the largest difference between PWS (mean of stuttering-biological and stuttering-psychological groups) and healthy adults. If the left side of the adjective pairs is selected, the response is the smaller number; if the right pair is selected, the response is the larger number.

**FIGURE 3:**
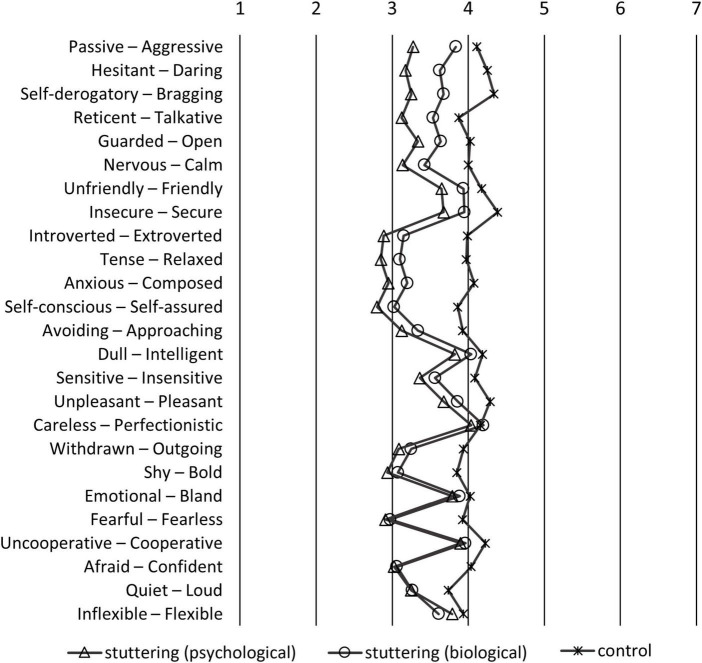
Means of the participants of the stereotypes questionnaire sorted by the largest difference between stuttering-biological and stuttering-psychological groups. If the left side of the adjective pairs is selected, the response is the smaller number; if the right pair is selected, the response is the larger number.

The mean of the questionnaire of stereotyped perception was calculated for the three groups (Figures 2, 3). For reverse scored items, the order of the bi-polar pairs was reversed. Figure 2 shows the items sorted from top to bottom; hence, the means of the stuttering-biological and stuttering-psychological groups are ordered according to the largest difference from the control group. The items at the top have a larger difference between the stuttering and control groups. For instance, the “tense-relaxed” pair was the most stereotyped item, where participants considered PWS as more “tense” compared to healthy adults. Similarly, participants considered PWS as more “anxious,” “afraid,” “fearful,” “introverted,” or “self-conscious.” In contrast, there is little or no difference between stuttering and control groups on the “inflexible-flexible,” “emotional-bland,” or “careless-perfectionistic” pairs.

The results shown in Figure 3 are similar to those in Figure 2, but are rearranged so that the difference in scores between stuttering-biological and stuttering-psychological groups is larger from top to bottom. The items at the top showed a larger difference between the stuttering-biological and stuttering-psychological groups. For instance, the “passive-aggressive” pair was the most stereotyped item. Participants who believed in the psychological model of stuttering considered PWS as more “passive” than those who believed in the biological model. Similarly, participants who believed in the psychological model considered PWS as more “hesitant,” “self-derogatory,” “reticent,” “guarded,” or “nervous.” In contrast, there was little or no influence on the “afraid-confident,” “quiet-loud,” or “inflexible-flexible” pairs.

### 3.3. Exploratory factor analysis of the questionnaire

Stereotyped perception items were summarized into a few concepts by factor analysis. A high KMO value (0.893) verified the items’ adequacy to be used in factor analysis. Among 25 items regarding stereotyped perception, a five-factor structure was adopted based on Kaiser (1960) criteria. Table 6 shows the factor analysis results of least-squares regression models with promax rotation. Factors were named based on the similarity of included items. Factor 1 (9 items), which consisted of pairs such as “introverted-extroverted,” was labeled “extroversion.” Factor 2 (7 items), which consisted of pairs such as “pleasant-unpleasant,” was labeled “comfort,” and Factor 3 (5 items), which consisted of pairs such as “aggressive-passive,” was labeled “expressive.” Factors 4 (2 items) and 5 (2 items) were not labeled, and excluded from further analysis as they only consisted of two items, and the reliability of internal consistency described in section “3.5. Reliability” was not confirmed.

**TABLE 6:** Factor analysis on the 25 items of the stereotypes questionnaire.

	Factor 1 “extroversion”	Factor 2 “comfort”	Factor 3 “expressive”	Factor 4	Factor 5	Communality
Introverted – Extroverted	**0.950**	0.109	−0.059	−0.152	0.092	0.696
Afraid – Confident	**0.877**	0.030	0.058	−0.197	−0.013	0.601
Fearful – Fearless	**0.868**	0.099	0.065	−0.098	0.028	0.565
Shy – Bold	**0.843**	0.081	−0.066	−0.015	0.102	0.628
Withdrawn – Outgoing	**0.721**	0.079	−0.093	−0.074	−0.066	0.517
Anxious – Composed	**0.719**	−0.160	0.209	−0.033	0.008	0.551
Self-conscious – Self-assured	**0.683**	0.038	0.011	0.103	−0.079	0.555
Tense – Relaxed	**0.655**	−0.148	0.223	0.103	−0.028	0.558
Avoiding – Approaching	**0.610**	−0.062	−0.172	−0.062	0.260	0.407
Pleasant – Unpleasant	0.102	**0.749**	0.208	−0.031	−0.190	0.526
Friendly – Unfriendly	0.058	**0.729**	0.115	−0.002	−0.137	0.489
Cooperative – Uncooperative	0.129	**0.694**	0.020	−0.003	−0.111	0.377
Secure – Insecure	−0.114	**0.569**	−0.015	0.067	−0.103	0.356
Intelligent – Dull	−0.089	**0.547**	0.042	0.341	0.017	0.486
Quiet – Loud	0.441	**0.473**	−0.337	0.267	0.094	0.498
Daring – Hesitant	−0.154	**0.400**	0.313	0.020	0.306	0.740
Aggressive – Passive	−0.087	0.127	**0.513**	−0.039	0.264	0.583
Talkative – Reticent	−0.141	0.194	**0.504**	−0.175	−0.067	0.452
Emotional – Bland	0.248	0.047	**0.464**	0.063	−0.044	0.213
Open – Guarded	−0.167	0.244	**0.359**	−0.075	0.054	0.399
Bragging – Self-derogatory	−0.188	0.291	**0.341**	−0.034	0.228	0.593
Careless – Perfectionistic	0.267	−0.178	0.111	**0.773**	0.037	0.545
Calm – Nervous	−0.341	0.359	−0.264	**0.436**	−0.163	0.595
Inflexible – Flexible	0.270	−0.274	0.155	0.059	**0.476**	0.292
Insensitive – Sensitive	−0.243	−0.286	−0.150	−0.287	**0.442**	0.401

Least-squares regression models with promax rotation.

### 3.4. Statistical comparison using ANOVAs

**FIGURE 4:**
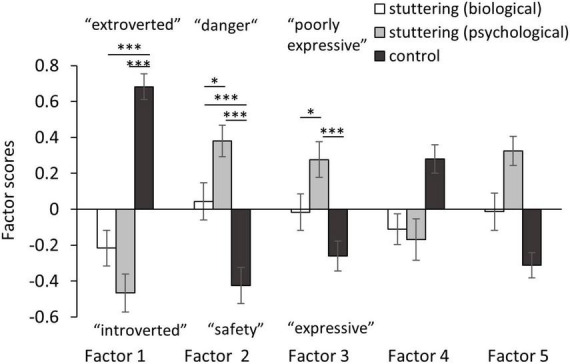
Mean factor scores of each characteristic among the three groups. Error bar represents standard deviation. ***p < 0.001, **p < 0.01, *p < 0.05.

Mean factor scores of each factor were then calculated (Table 7), with ceiling or floor effects not observed. ANOVAs of between-participant factors (stuttering-biological, stuttering-psychological, and control groups) and within-participant factors (characteristics) with Shaffer’s multiple comparisons were performed (Figure 4). Although there was no significant main effect of groups [F(1,32) = 1.97, *p* = 0.142, ηG2 = 0.004] and characteristics [F(1,32) = 0.00, *p* = 1, ηG2 = 0.000], significant interaction was observed between the two factors [F(1,32) = 22.78, *p* < 0.001, ηG2 = 0.125]. As there was a simple main effect of groups in all characteristics [F(2, 237) = 6.74∼41.74, *p* < 0.001, ηG2 = 0.05∼0.26], we made a *post-hoc* analysis to compare characteristics between each group. In the “extroversion” factor, compared to the control group, the stuttering-biological and stuttering-psychological groups significantly stereotyped PWS as “introverted” [biological: *t*(237) = 6.80, *p* < 0.001, *d* = 1.154; psychological: *t*(237) = 8.69, *p* < 0.001, *d* = 1.426], and there was no significant difference between the stuttering-biological and stuttering-psychological groups [*t*(237) = 1.89, *p* = 0.060, *d* = 0.273]. In the “comfort” factor, significant differences were observed among the three groups; compared to the control group, the stuttering-biological and stuttering-psychological groups significantly stereotyped PWS as “dangerous” [biological: *t*(237) = 3.39, *p* < 0.001, *d* = 0.513; psychological: *t*(237) = 5.83, *p* < 0.001, *d* = 0.953]. In addition, compared to the stuttering-psychological group, the stuttering-biological group regarded PWS as significantly less “dangerous” [*t*(237) = 2.44, *p* = 0.015, *d* = 0.393]. In the “expressive” factor, the stuttering-psychological group significantly stereotyped PWS as “poorly expressive” compared to the control group [*t*(237) = 3.98, *p* < 0.001, *d* = 0.654], and the stuttering-biological group significantly stereotyped PWS as “expressive” compared to the stuttering-psychological group [*t*(237) = 2.18, *p* = 0.031, *d* = 0.326]. There was no significant difference between the stuttering-biological and control groups [*t*(237) = 1.81, *p* = 0.072, *d* = 0.294].

**TABLE 7:** Mean, standard deviation, and range of participants regarding five characteristics factors.

	Stuttering (biological) group			Stuttering (psychological) group			Control group		
	Mean	Standard deviation	Range	Mean	Standard deviation	Range	Mean	Standard deviation	Range
Factor 1 “extroversion”	−0.22	0.90	−2.44–1.69	−0.47	0.94	−2.69–1.66	0.68	0.64	−2.21–2.01
Factor 2 “comfort”	0.04	0.93	−2.84–2.70	0.38	0.79	−1.73–3.77	−0.43	0.90	−3.27–2.47
Factor 3 “expressive”	−0.02	0.91	−2.73–3.26	0.28	0.89	−1.88–3.78	−0.26	0.75	−2.09–1.66
Factor 4	−0.11	0.76	−2.95–1.67	−0.17	1.03	−2.62–2.56	0.28	0.70	−1.69–2.35
Factor 5	−0.01	0.92	−3.59 – 2.00	0.33	0.73	−1.78–2.10	−0.31	0.64	−3.08–2.12

### 3.5. Reliability

Table 4 shows the internal consistency of each factor of stereotyped perception. Modest reliability of internal consistency was confirmed, which was 0.70 or higher (e.g., Tavakol and Dennick, 2011). Factors 4 (0.339) and 5 (0.142) were relatively low; this may be because only two items were included in each factor.

## 4. Discussion

### 4.1. Stereotyped perception of stuttering influenced by belief regarding its etiology

Items such as “tense,” “anxious,” “afraid,” “fearful,” “introverted,” or “self-conscious” (Figure 2) were considered stereotypes of PWS held by participants. In Woods and Williams’ (1976) study, participants regarded males who stutter as more “self-conscious,” “tense,” “guarded,” “withdrawn,” or “reticent” compared to males who do stutter. The item-by-item comparison of previous and present studies must be cautiously made because of confounding factors such as country or year of study. However, overall, our findings are consistent with previous studies, reflecting a biased perception of stuttering that is negatively stereotyped (e.g., St Louis and Tellis, 2015; Boyle, 2016a; Amick et al., 2017; Byrd et al., 2017). The strength of the present study is that it further elucidates the association between the implicit belief of the etiology of stuttering, and the negative perceptions of stuttering based on public opinion. Participants who believed in the psychological model held more stereotypes of PWS, such as “passive,” “hesitent,” “self-derogatory,” “reticent,” or “guarded,” compared to participants who believed in the biological model (Figure 3). Furthermore, factor analysis of the SD scales of stereotyped perception revealed that participants who believed in the biological model regarded stuttering males as “less dangerous” and “expressive” compared to participants who believed in the psychological model (Figure 4). Findings that the biological model was associated with less negative perceptions than the psychological model were consistent with previous research on stuttering (Boyle et al., 2009; Boyle, 2016a). While previous studies presented the etiology of stuttering explicitly by vignettes (Boyle et al., 2009; Boyle, 2016a), our study used the item scores after factor analysis of scales that asked about the belief of each participant, not explicitly about the “biological” or “psychological” models. Thus, our results could reflect participants’ implicit belief in the etiology of stuttering.

Our findings largely fall in line with previous studies in this area (Boyle et al., 2009; Boyle, 2016a); however, psychological impacts are complex in the literature on mental disorders. While some studies have supported the association of the biological model and positive attitude, such as increased empowerment, preference for psychotherapy, and decreased stigma (e.g., Goldstein and Rosselli, 2003), other empirical studies revealed opposite findings (Dietrich et al., 2004, 2006; Angermeyer and Matschinger, 2005; Bennett et al., 2008; Lincoln et al., 2008). Meta-analysis studies of 28 experimental mental disorders revealed that biogenetic explanations reduce blame, but induce pessimism, and increase endorsement of the stereotype that people with psychological problems are dangerous (Kvaale et al., 2013). The inconsistency also depends on the type of mental disorder (Dietrich et al., 2004; Breheny, 2007); etiology explanations work in complex ways and may not uniformly reduce illness-related stigma (Breheny, 2007). Breheny (2007) investigated the interaction of type of mental disorder and attitude; while an increase in willingness to interact was observed when schizophrenia was described as genetically caused, a reverse effect was observed in the case of major depression. In research on stuttering, no stigma-related scales showed any significant differences between biological and unknown explanations (Boyle et al., 2009), and biological and no explanations (Boyle, 2016a). Boyle (2016a,b) also stated that providing biological explanations for stuttering is not effective for reducing stigma compared to providing no explanation at all, and could increase prognostic pessimism. Our study could not draw a clear conclusion because we did not use vignettes of stuttering to provide stuttering information. However, keeping in mind that the general public’s belief that the etiology of stuttering is psychological (e.g., Van Borsel et al., 1999; Ming et al., 2001; de Britto Pereira et al., 2008; St Louis, 2011; Abdalla and St Louis, 2012; Iimura et al., 2018), an anti-stigma campaign to remove this negative stereotyped perception should be promoted.

### 4.2. Implications for anti-stigma campaigns

To prevent prejudice or discrimination caused by the misunderstanding of stuttering, the importance of public education and campaigns to improve knowledge regarding stuttering should be highlighted. As Boyle et al. (2009) stated, effective anti-stigma campaigns might be valuable in decreasing public stigma associated with stuttering. Among mental disorders, such as schizophrenia and depression, three approaches to change [i.e., education, contact, and protest (Corrigan and Penn, 1999; Corrigan et al., 2012)] have been explored; both education and contact have had a positive effect on reducing stigma (Corrigan et al., 2012). Boyle et al. (2016) applied three anti-stigma strategies to research on stuttering, and revealed that all were effective in reducing stereotypes, negative emotions, and discriminatory intentions; contact had the most positive effect.

Our findings can contribute toward future anti-stigma campaigns (e.g., Boyle et al., 2016, 2017; Boyle, 2017). The contact approach can use messages that describe the challenges of stuttering and the recovery process, in addition to a clear request to promote positive attitudes and behaviors toward PWS (Boyle et al., 2017). Informing the general public that stuttering cannot be voluntarily eliminated may also prove effective. Previous research found that contact experience or familiarity with stuttering can improve people’s negative attitudes (e.g., Flynn and St Louis, 2011; Abdalla and St Louis, 2012; Arnold and Li, 2016; Boyle et al., 2016, 2017; Hughes et al., 2017; Iimura and Miyamoto, 2021). Education or protest approaches could be helpful in providing a more accurate understanding of stuttering by offering facts and dispelling myths about stuttering, emphasizing that there are successful PWS who have jobs that require speaking, and stating that PWS are fundamentally no different from other people despite their disfluent speech (Boyle et al., 2017). Education about stuttering is also effective in preventing bullying against children who stutter (Langevin and Prasad, 2012). Through these anti-stigma strategies, the public’s beliefs regarding stuttering can be changed, thereby decreasing the emotional response and discrimination and increasing helping behavior (Corrigan et al., 2003).

The type of information regarding stuttering etiology applicable toward anti-stigma campaigns has some limitations, and this could limit our implications. Our focus is the psychological and biological models of stuttering, but stuttering is considered to be a multi-dimensional and multi-factorial set of causes; thus, it is unclear whether our study’s assumption is justified by the etiology of stuttering research. Recent genetic or neuroimaging approaches have revealed the genetic basis for the occurrence of stuttering and its predominantly biological roots (e.g., Kraft and Yairi, 2012; Chang and Zhu, 2013; Frigerio-Domingues and Drayna, 2017; Guitar, 2018). However, not all causes are explained by these biological roots, and are likely to be heterogenous, involving a combination of biological, psychological, and social factors (e.g., Smith and Weber, 2017). Future advancement of the etiology of stuttering could justify the rationale of our study.

While the questionnaire regarding the etiology of stuttering (section “2.3.1. Belief regarding the etiology of stuttering”) included items that are supposed to be “environmental,” we did not consider the biopsychosocial framework, such as the International Classification of Functioning, Disability, and Health (ICF), for the included items. In the ICF framework, disability is illustrated as the interaction of personal conditions and social-related factors such as personal and environmental factors (World Health Organization [WHO], 2001; Yaruss and Quesal, 2004; Tichenor and Yaruss, 2019). Furthermore, the framework of the ICF illustrates not only the causes of disabilities, but also their effects, such as how the reactions speakers receive from other people can influence their own reactions to stuttering (Yaruss and Quesal, 2004; Tichenor and Yaruss, 2019). The social factors of stuttering should be assessed in future research.

It is obvious that a gap exists between public belief and recent empirical findings. Previous studies on public knowledge regarding stuttering reveal that the majority of the general population considers stuttering to be caused by a psychological problem, that is, “psychogenic” (Van Borsel et al., 1999; Iimura et al., 2018) or “emotional” (de Britto Pereira et al., 2008), rather than neurological, genetic, or familial factors. However, neither the multifactorial causes theory nor the biological roots of stuttering theory support the theory of it being caused by a psychological factor alone. Boyle (2016b) prospects future implications based on the presumption that it is an oversimplification to discuss a single cause of stuttering in isolation, and that using a multidimensional explanation may be the most effective approach for reducing stigma. Therefore, although simplification of the biological model is not recommended, the psychological model, which is the model the general public is mostly likely to believe, should educate the public by presenting the causes of stuttering as being related in a multidimensional framework with a genetic basis and brain structural/functional abnormalities, and that psychological factors are also involved in the persistence of stuttering or in worsening the psychological impact.

### 4.3. Limitations and implications for further study

In the present study, a detailed description of stuttering, such as a vignette, was not provided to the participants; hence their responses regarding perceptions of stuttering could have been influenced by their current or past knowledge or contact with PWS. Hence, it is desirable to control these factors in future research.

Perception ratings in the present study were focused on males who stutter and healthy adult males. As stuttering is typically more prevalent in males than females, our results could be representative of the majority of PWS. However, to generalize the study results, it is necessary to consider the participants’ gender.

As elaborated in section “4.2. Implications for anti-stigma campaigns,” it is unclear whether the biological model of the etiology of stuttering helps decrease the negative stereotyped perception of stuttering compared to providing no additional information or an unknown explanation of the etiology. In the present study, the grouping of etiology of stuttering was based on participants’ beliefs, and the control condition of assessing PWS could not be included in this study design. Further investigations should elaborate the appropriate ways to promote anti-stigma campaigns. Again, the implications of our study findings are preliminary for developing an anti-stigma campaign because we did not consider the biopsychosocial framework for the etiology of stuttering. Stigmatization could be affected by not only the social perception of the etiology of stuttering but also other factors, such as social stereotypes or misunderstanding of stuttering. Further research that considers social-related factors to be the cause of stuttering are needed to understand the interaction of this multifactorial disorder in the biopsychosocial framework.

## 5. Conclusion

The present study is the first to investigate the association between implicit belief regarding the etiology of stuttering and its negative perceptions. Participants who believed in the biological model had a less negative perception of stuttering than those who believed in the psychological one. Our study’s findings contribute to the anti-stigma campaign of stuttering with regard to how the etiology of stuttering is described.

## Data availability statement

The raw data supporting the conclusions of this article will be made available by the authors, without undue reservation.

## Ethics statement

The studies involving humans were approved by the Kawasaki University of Medical Welfare. The studies were conducted in accordance with the local legislation and institutional requirements. The participants provided their written informed consent to participate in this study.

## Author contributions

DI: Conceptualization, Data curation, Funding acquisition, Investigation, Methodology, Project administration, Validation, Writing – original draft, Writing – review and editing. OI: Conceptualization, Writing – original draft, Writing – review and editing.

## Footnotes

1
www.ulatus.jp
