# Supplementary material for: Comparing the beliefs regarding biological or psychological causalities toward stereotyped perception of people who stutter
Source: Front Psychol. 2023 Nov 16;14:1279169. doi: 10.3389/fpsyg.2023.1279169 (PMC10687552; doi:10.3389/fpsyg.2023.1279169)
Supplement: Supplementary file 1 [file Table_1.DOCX]

Appendix. Questionnaire used in the present study

1. Please select whether a given factor was a cause of stuttering (“certainly not a cause (scored 1)” to “certainly a cause (scored 7)”)

biochemical abnormalities

biological changes in brain

biology

brain damage (e.g., poisoning or injuries)

brain disease (transmitter disorder and morphological anomalies)

chemical/ hormone imblance

emotional

environmental factors

expecting too much of self

faulty learning or habits

general stress

ghosts, demons, spirits

God’s will (e.g., punishment or test)

inheritance/genetics

lack of will power

learned helplessness

low (lack of) social support

melancholic personality

negative life event

personality

poor cognitive outlook

problematic childhood (e.g., unloving parents, too strict, or inconsequent upbringing)

psychogenic

recent misfortunes

self-induced (e.g., weak will, impulsiveness, or immoral behavior)

stressors and strain

traumatic event

(*The order of items was randomized across participants.)

1. Please select from the pairwise combinations what you believe describes an average, typical stuttering male adult.

Open – guarded

Nervous – calm

Cooperative – uncooperative

Shy – bold

Friendly – unfriendly

Self-conscious – self-assured

Tense – relaxed

Sensitive – insensitive

Anxious – composed

Pleasant – unpleasant

Withdrawn – outgoing

Quiet – loud

Intelligent – dull

Talkative – reticent

Avoiding – approaching

Fearful – fearless

Aggressive – passive

Afraid – confident

Introverted – extroverted

Daring – hesitant

Secure – insecure

Emotional – bland

Perfectionistic – careless

Bragging – self-derogatory

Inflexible – flexible

(*The order of items was randomized across participants.)

1. Please select from the pairwise combinations what you believe describes an average, typical male adult.

Open – guarded

Nervous – calm

Cooperative – uncooperative

Shy – bold

Friendly – unfriendly

Self-conscious – self-assured

Tense – relaxed

Sensitive – insensitive

Anxious – composed

Pleasant – unpleasant

Withdrawn – outgoing

Quiet – loud

Intelligent – dull

Talkative – reticent

Avoiding – approaching

Fearful – fearless

Aggressive – passive

Afraid – confident

Introverted – extroverted

Daring – hesitant

Secure – insecure

Emotional – bland

Perfectionistic – careless

Bragging – self-derogatory

Inflexible – flexible

(*The order of items was randomized across participants.)
